# MicroRNAs regulate key cell survival pathways and mediate chemosensitivity during progression of diffuse large B-cell lymphoma

**DOI:** 10.1038/s41408-017-0033-8

**Published:** 2017-12-15

**Authors:** Suvi-Katri Leivonen, Katherine Icay, Kirsi Jäntti, Ilari Siren, Chengyu Liu, Amjad Alkodsi, Alejandra Cervera, Maja Ludvigsen, Stephen Jacques Hamilton-Dutoit, Francesco d’Amore, Marja-Liisa Karjalainen-Lindsberg, Jan Delabie, Harald Holte, Rainer Lehtonen, Sampsa Hautaniemi, Sirpa Leppä

**Affiliations:** 1 Research Programs Unit, Genome-Scale Biology, Faculty of Medicine, University of Helsinki, Helsinki, Finland; 2 Department of Oncology, Helsinki University Hospital Cancer Center, Helsinki, Finland; 3 Department of Clinical Medicine, Aarhus University, Aarhus, Denmark; 4 Department of Hematology, Aarhus University Hospital, Aarhus, Denmark; 5 Institute of Pathology, Aarhus University Hospital, Aarhus, Denmark; 6 Department of Pathology, Helsinki University Hospital, Helsinki, Finland; 7 Department of Pathology, University of Toronto, Toronto, ON Canada; 8 Department of Oncology, Oslo University Hospital, Oslo, Norway

## Abstract

Despite better therapeutic options and improved survival of diffuse large B-cell lymphoma (DLBCL), 30–40% of the patients experience relapse or have primary refractory disease with a dismal prognosis. To identify biological correlates for treatment resistance, we profiled microRNAs (miRNAs) of matched primary and relapsed DLBCL by next-generation sequencing. Altogether 492 miRNAs were expressed in the DLBCL samples. Thirteen miRNAs showed significant differential expression between primary and relapse specimen pairs. Integration of the differentially expressed miRNAs with matched mRNA expression profiles identified highly anti-correlated, putative targets, which were significantly enriched in cancer-associated pathways, including phosphatidylinositol (PI)), mitogen-activated protein kinase (MAPK), and B-cell receptor (BCR) signaling. Expression data suggested activation of these pathways during disease progression, and functional analyses validated that miR-370-3p, miR-381-3p, and miR-409-3p downregulate genes on the PI, MAPK, and BCR signaling pathways, and enhance chemosensitivity of DLBCL cells in vitro. High expression of selected target genes, that is, *PIP5K1* and *IMPA1*, was found to be associated with poor survival in two independent cohorts of chemoimmunotherapy-treated patients (*n* = 92 and *n* = 233). Taken together, our results demonstrate that differentially expressed miRNAs contribute to disease progression by regulating key cell survival pathways and by mediating chemosensitivity, thus representing potential novel therapeutic targets.

## Background

Diffuse large B-cell lymphoma (DLBCL) is the most common lymphoid malignancy in adults. It is a heterogeneous disease, which can be classified into activated B-cell, germinal center B-cell (GCB), and primary mediastinal B-cell subtypes according to gene expression profiling^1–4^. The standard therapy for DLBCL is a combination of CD20 antibody rituximab with cyclophosphamide, doxorubicin, vincristine, and prednisone (R-CHOP)^5, 6^. Despite the efficacy of this regimen, approximately one-third of the patients have primary refractory disease or relapse, which remains a major cause of morbidity and mortality. However, molecular mechanisms behind the treatment failure remain largely unknown.

MicroRNAs (miRNAs) participate in several biological processes by regulating gene expression at the posttranscriptional level. These short non-coding RNAs (20- to 22-nucleotides) bind to complementary sites in their target gene mRNAs, thereby inhibiting translation or inducing destabilization and degradation of the target mRNAs^7^. Moreover, dysregulation of miRNAs has been linked to the development and progression of a number of human cancers, making them ideal candidates for both predictive and prognostic biomarkers^8, 9^.

Recently, an 8-miRNA classifier was reported to distinguish among DLBCL molecular subgroups^10^. MiRNA signatures can also identify specific tumor drug resistances or drug sensitivities, and predict clinical outcome in DLBCL patients treated with chemoimmunotherapy^10–12^. A comprehensive profiling of the DLBCL miRNome identified miRNAs associated with patient survival independently of established indicators of outcome^13^. MiRNAs, such as miR-155, miR-21, and members of the miR-17-92 cluster have been demonstrated to drive lymphomagenesis in miRNA mouse models^14–18^, while the miR-34 family represents a tumor suppressor in DLBCL^19^. However, so far, the potential regulatory role of miRNAs in DLBCL progression has not been explored.

Here, we have performed miRNA and mRNA profiling of matched primary and relapsed DLBCLs. Our work establishes a landscape of miRNA expression in poor prognosis DLBCL, and highlights regulatory roles for miRNAs in signaling pathways contributing to disease progression and poor survival.

## Methods

### Patient samples

The discovery cohort consisted of matched primary-relapse sample pairs from seven DLBCL patients (Supplementary Table 1). The patient selection was based on availability of fresh frozen tissue containing adequate material for RNA extraction and next-generation sequencing (NGS). The patients were 62–76 years old and had a primary, high-risk (age-adjusted International Prognostic Index (aaIPI) score 2–3) disease. They had received chemoimmunotherapy (R-CHOP or R-CHOEP), but relapsed between 4 to 62 months after the treatment.

The validation cohort consisted of 13 primary-relapse sample pairs from formalin-fixed paraffin-embedded tissue containing adequate material for RNA extraction for real-time reverse transcription PCR (qRT-PCR) (Supplementary Table 1). The patients were 44–81 years old and had a primary, high-risk disease. They had received chemoimmunotherapy (R-CHOP or R-CHOEP), but relapsed between 4 to 157 months after the treatment.

The study was approved by the National Authority for Medicolegal Affairs, Finland, and Institutional Review Boards, and Ethics Committees in Helsinki, Finland, Aarhus, Denmark and Oslo, Norway. All patients gave informed consent.

### Next-generation sequencing and data analysis

RNAs from seven primary-relapse DLBCL sample pairs (Supplementary Table 1) were subjected to NGS. Expression quantification was performed for all but one relapse sample (DLBCL1_R), which was filtered out during quality control. TruSeq small RNA sample preparation kit (Illumina, Inc. CA, USA) was used for library construction according to manufacturer’s protocol. Cluster generation and sequencing were performed on the Illumina HiSeqTM 2000 platform by Beijing Genomics Institute (Beijing, China), with average read depth of ~ 30 M per sample. More details on data analysis are provided in the Supplementary methods. The NGS data have been submitted to the Gene Expression Omnibus (GEO) database with accession number GSE69810.

### miRNA−mRNA transcript target pair analysis

We identified highly anti-correlated (Spearman rho < −0.7, *p* < 0.05) transcript expression across the samples for each miRNA. Anti-correlated miRNA−mRNA pairs were then filtered for support by at least one of the selected target database resources (TargetScan Human v.5.2^20^, DIANA microT v.18^21^, Microcosm v.5^22^, PITA v.5^23^, and a manually curated mirTarbase v.4.5^24^). The miRNA-target gene pairs then proceeded to gene ontology (GO) and KEGG pathway enrichment analysis.

### Real-time reverse transcription PCR (qRT-PCR) analysis

MiRNA expression was analyzed with the miRCURY LNA™ Universal RT microRNA PCR system (Exiqon, Vedbaek, Denmark) using 100 ng RNA as starting material. Relative expression was determined by the 2^−ΔΔC^_T_ method^25^ with 5S RNA as an endogenous control. The samples were run at least twice in triplicates.

For determining the target gene mRNA expression, 1 µg total RNA was reverse transcribed with iScript cDNA synthesis kit (Bio-Rad Laboratories Inc., Hercules, CA, USA). Subsequently, the cDNAs were diluted 1/10 and subjected to Taqman Fast qPCR with Gene Expression Assays from Applied Biosystems. Relative expression was determined by the 2^−ΔΔC^_T_ method^25^ using GAPDH as an endogenous control. The samples were run three times with triplicates.

### Survival analyses and statistics

For survival analyses, data from the Cancer Genome Characterization Initiative (CGCI) (*n* = 92) (the database of Genotypes and Phenotypes study accession: phs000532.v2.p1)^26, 27^ and the Lymphoma/Leukemia Molecular Profiling Project (LLMPP) (*n* = 233) (GEO accession: GSE10846)^2^ were used. CGCI level 1 RNA-seq data were downloaded and processed with SePIA transcriptomics analysis pipeline^28^. A web-based cutoff finder tool (http://molpath.charite.de/cutoffanalysis) was used to determine the most prognostic cutoff level for survival outcomes^29^. Kaplan−Meier plots were created with SPSS 22.0 (IBM, Armonk, NY, USA) and log-rank test was used for calculating the significance. Overall survival (OS) was determined from the date of diagnosis until last follow-up or death from any cause. Progression-free survival (PFS) was measured as the period between the date of diagnosis and progression or death from any cause. Multivariate analyses were performed according to the Cox proportional hazards regression model using categorical data. *p*-values < 0.05 were considered significant and all *p*-values were two-sided.

### Cell culture and lentiviral transductions

SU-DHL-4 cells were from Deutsche Sammlung von Microorganismen und Zellkulturen GmbH (DSMZ) and they were cultured in RPMI 1640 Medium (Corning Life Sciences, Tewksbury, MA, USA) in the presence of 10% fetal bovine serum, 2 mm l-glutamine and 1% penicillin/streptomycin. The cells were tested and authenticated by Short Tandem Repeat profiling and checked for mycoplasma infections regularly using the MycoAlert™ Mycoplasma Detection Kit (Lonza, Basel, Switzerland).

The human shMIMIC lentiviral vectors for miR-370-3p, miR-381-3p, and miR-409-3p were obtained from GE Dharmacon (Lafayette, CO). SmartVector shMIMIC non-targeting Control 1 (Co1) and Control 10 (Co10) were used as negative controls. The lentiviral vectors had hEF1 promoter and expressed turboGFP. SU-DHL-4 cells were transduced with the lentiviral vectors and incubated for three days. Thereafter, the cells were subjected for puromycin (1 µg/ml) selection to create stable cell lines. The GFP and miRNA expression of the cell lines was confirmed by microscopy and qRT-PCR, respectively.

### Cell viability assays

SU-DHL-4 cells stably expressing miR-370-3p, miR-381-3p, and miR-409-3p as well as non-targeting control Co10 were plated on black, clear-bottom 96-well plates (25,000 cells/well). Rituximab (1 µg/ml) or doxorubicin (100 nm) was added at the time of plating. The cells were incubated for 72 h, and cell viability measured with CellTiter-Blue Cell Viability assay (Promega, Madison, WI). The experiments were done in triplicates and repeated for four times.

## Results

### MiRNA sequencing of primary and relapsed DLBCL

**Fig. 1:**
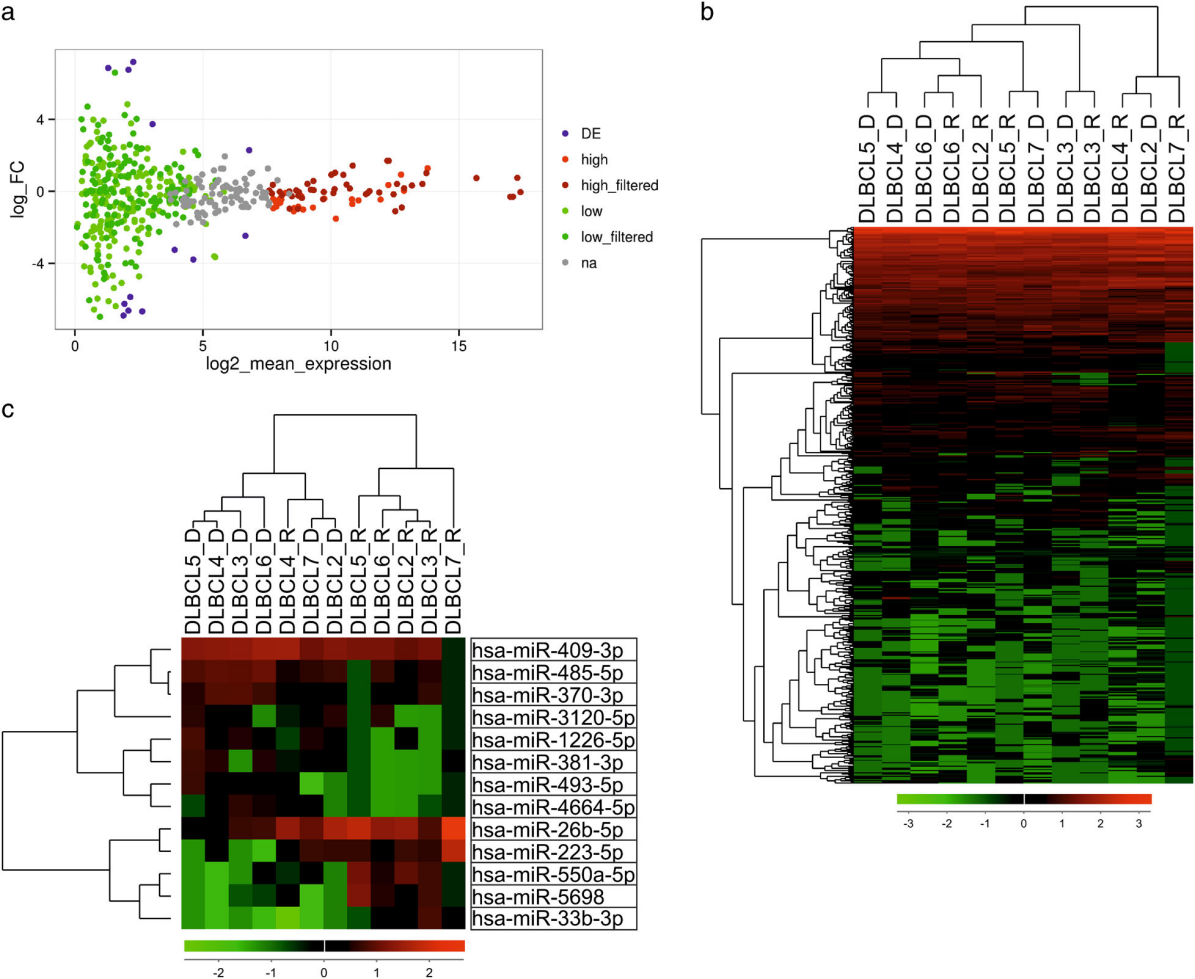
MiRNA-sequencing results Seven primary-relapse DLBCL sample pairs were subjected to next-generation miRNA sequencing. One relapse sample (DLBCL1_R) was exluded from the analyses because it failed the quality control. Therefore, the paired analyses included data from six pairs. **a** MA (Log ratio (M) vs. mean average (A) expression) plot visualizing miRNA expression in primary and relapse sample pairs. The colors denote different subgroups of miRNAs: differentially (DE), high and low expressed (high and low expressed miRNAs were filtered against a reference data set from normal and non-malignant B-cells, as described in Methods). **b** A heatmap visualizing clustering of the DLBCL samples based on their miRNA expression. **c** A heatmap of miRNAs differentially expressed between primary and relapse pairs with *p*-value < 0.05. FC fold change, na not assigned

To uncover molecular mechanisms behind treatment resistance and progression in DLBCL, we searched for differentially expressed miRNA profiles between matched primary and relapsed DLBCLs. The discovery cohort consisted of seven DLBCL patients (Supplementary Table 1). A total of 492 known miRNAs were detected in the samples (Fig. 1a, b). Overall, the miRNA expression profiles in the primary and relapse samples were quite similar, suggesting that the miRNA expression remains relatively constant during the disease progression (Fig. 1a). Unsupervised clustering analysis of the miRNA expression could not distinguish primary from relapsed tumor samples (Fig. 1b).

We identified subsets of miRNAs with high (*n* = 24) and low expression (*n* = 177) as compared to a reference set of non-malignant B-cells (Fig. 1a; Supplementary Table 2). Among the high-expressed miRNAs were members of the miR-10 family, which has been demonstrated to have oncogenic effects for many cancer cells^30^. In contrast, many low-expressed miRNAs, including miR-129-5p^31, 32^, miR-663^33, 34^, and miR-203a^35, 36^, are known from their tumor suppressive roles. Further analysis revealed hypermethylation in the promoter regions of these tumor-suppressive miRNAs (Supplementary Fig. 1), suggesting methylation as the cause for their downregulation in DLBCL.

The analysis revealed 13 miRNAs with differential expression (*p* < 0.05) between primary and relapsed samples. Five miRNAs had higher and eight had lower expression in the relapse samples as compared to the primary samples (Fig. 1c and Supplementary Table 3).

### MiRNA−mRNA data integration reveals potential miRNA target genes enriched in lymphoma-associated pathways

Given that miRNAs often regulate target gene expression by inducing RNA degradation^37^, identification of negatively correlated miRNA and mRNA transcript interactions provides functional insights to the oncogenic mechanisms of miRNAs. Therefore, we correlated miRNA expression with that of mRNAs derived from total RNA-sequencing data, and subsequently integrated these with miRNA target predictions. For the high expressed miRNAs, the analysis resulted in 243 miRNA-transcript pairs representing 186 individual genes (Supplementary Table 4). GO enrichment analysis showed these genes to be enriched for cell adhesion (not shown). Altogether 5531 miRNA−mRNA transcript pairs for the low expressed miRNAs were identified, and they represented 3064 individual genes (Supplementary Table 5). These genes were enriched for cancer-related pathways or processes, such as KEGG-pathways in cancer, MAPK signaling pathway, cell cycle and apoptosis (Supplementary Table 6), suggesting that the low-expressed miRNAs contain tumor suppressive miRNAs, and their oncogenic targets are over-expressed in the DLBCL.

**Fig. 2:**
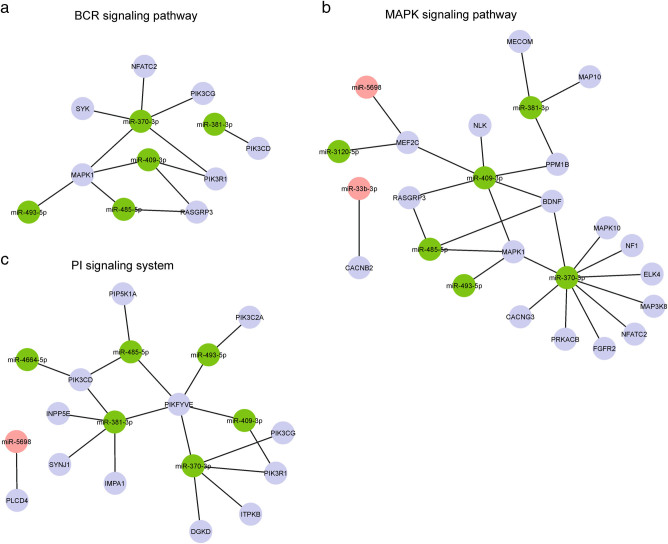
Pathways regulated by the differentially expressed miRNAs **a**–**c** The networks visualize miRNA-target gene associations with the B-cell receptor signaling (**a**), MAPK signaling (**b**), and phosphatidylinositol signaling system (**c**). Green circles denote miRNAs downregulated in relapse, whereas red circles denote miRNAs upregulated in relapse as compared to primary tumor. The networks were created with Cytoscape 3.1.1

The number of miRNA−mRNA pairs for the 13 differentially expressed miRNAs was 1088 representing 787 individual genes (Supplementary Table 7). These were enriched for lymphoma-associated pathways^38, 39^, including PI signaling system (e.g. *IMPA1, PIP5K1A, PIK3C2A, PIK3CG, PIK3R1*), JAK-STAT cascade (e.g. *STAT5A, STAT5B*), BCR signaling (e.g. *SYK, MAPK1, PIK3R1, PIK3CG, PIK3CD, RASGRP3*) and MAPK signaling (e.g. *MAPK1, MAPK10, MAP3K8, CACNG3*) (Supplementary Table 8). Interestingly, these pathways were linked to the targets of those differentially expressed miRNAs that showed lower expression in relapse samples (Fig. 2 and Supplementary Fig. 2).

### Validation of the differentially expressed miRNAs

**Fig. 3:**
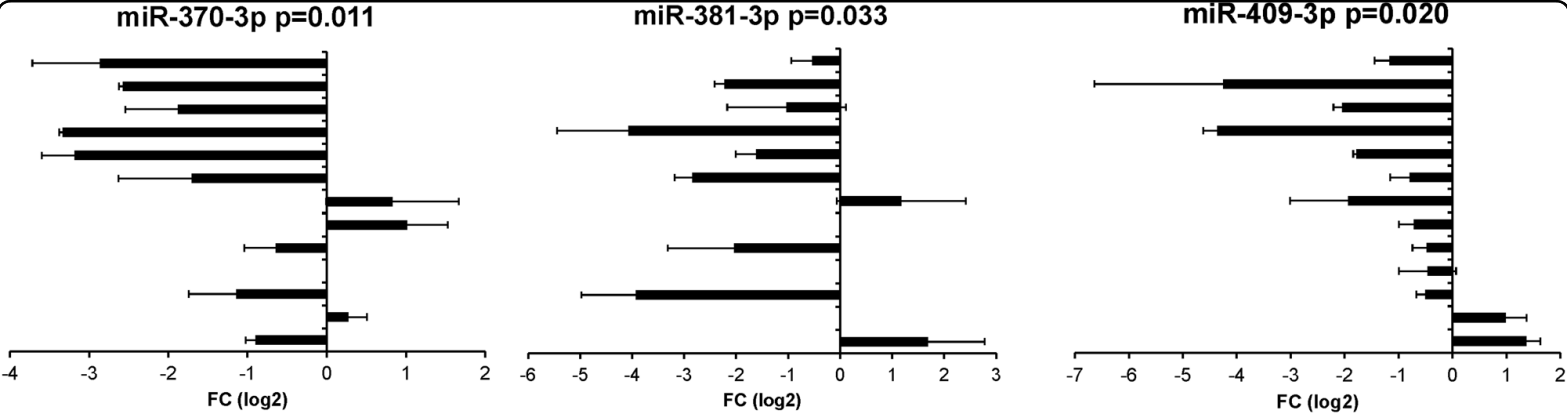
Validation of differentially expressed miRNAs MiRNAs were validated in a cohort of 13 FFPE sample pairs with the miRCURY LNA™ Universal RT microRNA PCR system using 5S RNA as an endogenous control. MiRNAs which were significantly differentially expressed are shown in the figure. Mean±SD of log2 transformed relative expression values in primary vs. relapse samples are shown (negative values, down in relapse; positive values, up in relapse; zero values, no expression). All samples were run at least twice in triplicates

To validate the NGS data, we performed qRT-PCR for eight differentially expressed miRNAs (miR-409-3p, miR-381-3p, miR-493-5p, miR-370-3p, miR-4664-5p, miR-485-5p, miR-26b-5p, and miR-550-5p) using an independent set (*n* = 13) of matched primary and relapsed DLBCL samples. Five miRNAs were excluded from the validation, because of low expression levels undetectable by qRT-PCR or because no functional primer pairs were available. In the validation set, three miRNAs (miR-409-3p, miR-381-3p, and miR-370-3p) were significantly downregulated in majority of the relapse samples (Fig. 3). The results provide support for the NGS results, and confirm the low expression of these miRNAs in relapsed DLBCL.

### Overexpression of miR-370-3p, miR-381-3p, and miR-409-3p increases sensitivity to rituximab and doxorubicin

**Fig. 4:**
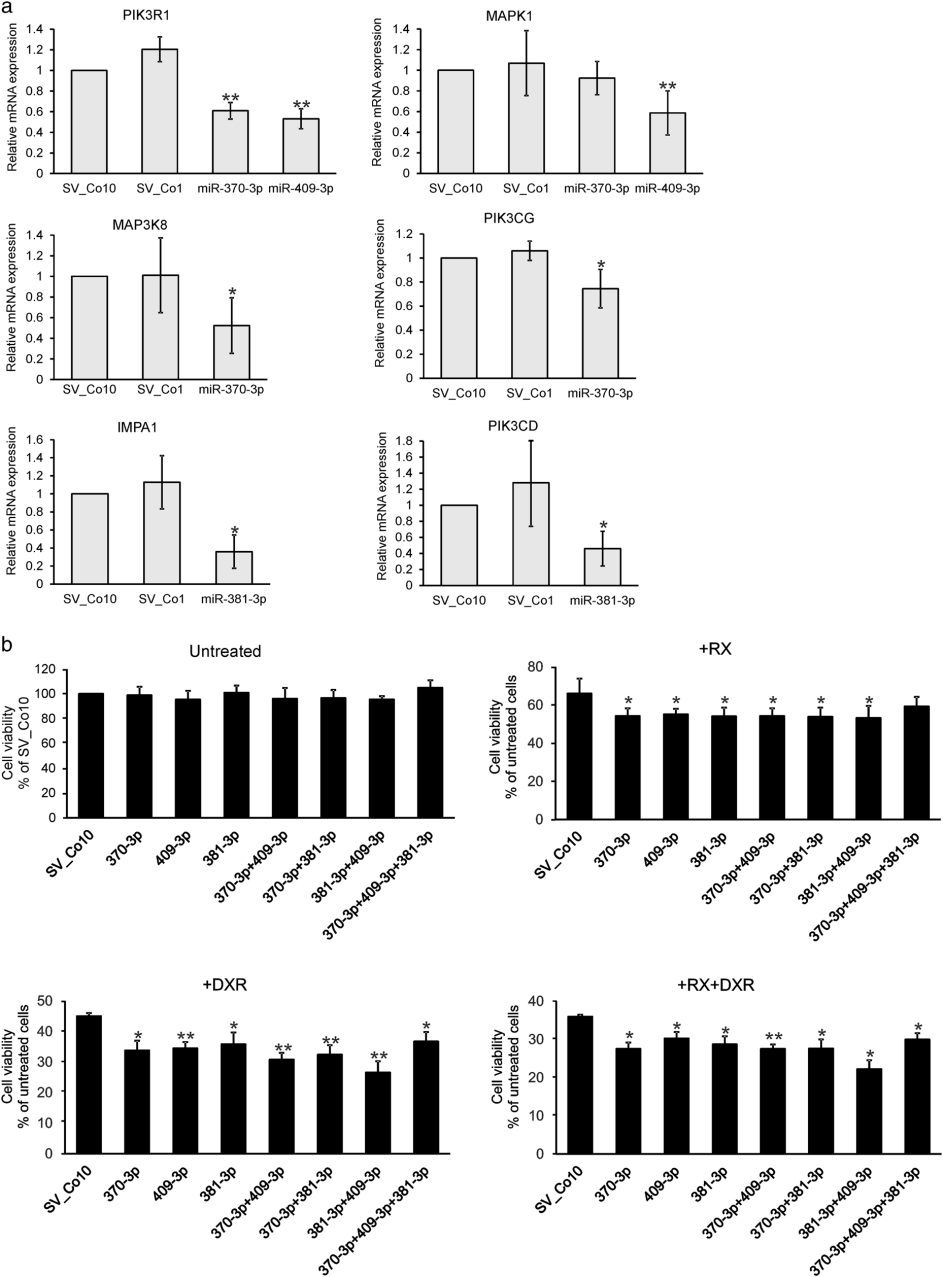
MiRNA-mediated regulation of target gene expression and drug response **a** The expression of putative miRNA target genes was determined by qRT-PCR from the mRNAs isolated from SU-DHL-4 cells stably overexpressing miR-370-3p, miR-381-3p, or miR-409-3p. Cells expressing non-targeting controls (Co1 or Co10) were used as negative controls. The results normalized for GAPDH are shown as relative expression (2^^−ΔΔCt^). The samples were run three times with triplicates, and the results are shown as mean±SD. **b** SU-DHL-4 cells stably overexpressing miR-370-3p, miR-381-3p, or miR-409-3p were treated with rituximab (RX; 1 µg/ml), doxorubicin (DXR; 100 nm), as indicated. After 72 h incubation, the cell viability was determined by CellTiter-Blue Cell Viability assay. Non-targeting SV_Co10-expressing cells were used as controls. The experiment was performed in triplicates and repeated four times. The results are shown as mean±SEM. *p < 0.05, **p < 0.01

In functional analyses we chose to focus on miR-370-3p, miR-381-3p, and miR-409-3p as these differentially expressed miRNAs showed significantly lower expression in the relapsed samples, and appeared to be the most potent regulators of the MAPK, BCR, and PI signaling system. MiR-370-3p, miR-381-3p, and miR-409-3p were stably overexpressed in SU-DHL-4 cells using lentiviral vectors (Supplementary Fig. 3), and the expression of selected target genes from the MAPK, BCR, and PI signaling system was assayed by qRT-PCR. Overexpression of miR-370-3p resulted in downregulation of MAP3K8, PIK3R1, and PIK3CG mRNA (Fig. 4a). MiR-409-3p downregulated PIK3R1 and MAPK1 mRNA, whereas miR-381-3p suppressed IMPA1 and PIK3CD mRNA (Fig. 4a). MiRNA-induced regulation was detected also at the protein level, where miR-370-3p and miR-409-3p inhibited ERK1/2 protein levels (Supplementary Fig. 4). MiR-370-3p decreased PIK3CG protein levels, which is in line with the in silico and qRT-PCR results. However, also miR-409-3p and miR-381-3p affected PIK3CG, which is not predicted to be targeted by these miRNAs. This highlights the importance of experimental validation and verifies the well-known fact that miRNAs have a broad impact on protein expression^40^.

Next, we examined whether the miRNAs enhance the growth inhibitory effects of doxorubicin and rituximab, the key components of the R-CHOP regimen. In untreated cells, the miRNA overexpression did not affect the cell growth (Fig. 4b). However, when the cells were treated with rituximab or doxorubicin alone or in combination, the number of viable cells was lower in the miRNA-expressing cells, as compared to control SV-Co10 cells (Fig. 4b). The data suggest that miR-370-3p, miR-381-3p, and miR-409-3p favor a response of DLBCL cells to rituximab and doxorubicin, and thus are involved in the mechanisms of chemosensitivity or -resistance.

### Survival association of the target genes

We investigated whether the expression of the target genes of the differentially expressed miRNAs is associated with survival. First, the prognostic impact was assessed in the CGCI cohort of 92 patients treated with R-CHOP-like regimen^26^. Clinical characteristics of the patients are shown in Table 1. Twenty-eight genes from the PI signaling system, BCR, and MAPK signaling pathways targeted by the differentially expressed miRNAs were used in the survival analyses. In Kaplan−Meier analyses, six genes were associated with survival (Table 2 and Supplementary Fig. 4). The patients with high expression of *SYK, MAPK1, CACGN3, IMPA1, PIP5K1A*, and *RASGRP3* genes had a shorter survival in comparison to the remaining patients with lower mRNA levels. In Cox multivariate analyses with IPI, *PIP5K1A* expression remained an independent negative prognostic factor for both PFS (RR = 3.431, CI95 = 1.407–8.366, *p* = 0.007) and OS (RR = 3.897, CI95 = 1558–9.744, *p* = 0.004), whereas *IMPA1* and *RASGRP3* had adverse prognostic impact for OS (RR = 2.850, CI95 = 1.202–6.757, *p* = 0.017 and RR = 3.270, CI95 = 1.255–8.520, *p* = 0.015, respectively), and *SYK* for PFS (RR = 2.925, CI95 = 1.152–7.425, *p* = 0.024) (Supplementary Fig. 5). When target gene-related PFS and OS were analyzed separately for the patients in different molecular subtypes, high expression of *PIP5K1A* and *RASGRP3* was associated with shorter survival in the non-GCB subtype, whereas high expression of *IMPA1* was associated with shorter survival in the GCB subtype. *SYK* was associated with shorter PFS in the GCB subtype, and *MAPK1* and *CACNG3* were associated with shorter PFS in the non-GCB subtype (data not shown). When clinical characteristics and gene expression were compared according to molecular subtypes, no differences were observed between the subgroups (Table 1).

**Table 1:** Patient characteristics of the CGCI and LLMPP cohorts

		CGCI				LLMPP			
		**Patients** **n (%)**	**GCB** **n (%)**	**Non-GCB** **n (%)**	***p*** **-val**	**Patients** **n (%)**	**GCB** **n (%)**	**Non-GCB** **n (%)**	***p*** **-val**
	All *n* (%)	92 (100)	51 (55)	41 (45)		233 (100)	107 (46)	126 (54)	
Gender	Female	31 (34)	19 (37)	12 (29)	0.51	99 (42)	49 (46)	50 (40)	0.356
	Male	61 (66)	32 (63)	29 (71)		134 (58)	58 (54)	76 (60)	
Age	<60	39 (42)	20 (39)	19 (46)	0.78	109 (47)	56 (52)	53 (42)	0.264
	60–65	15 (16)	9 (18)	6 (15)		29 (12)	13 (12)	16 (13)	
	>60	38 42)	22 (43)	16 (39)		95 (41)	38 (36)	57 (45)	0.109
Stage	I−II	44 (48)	28 (55)	16 (39)	0.15	105 (45)	54 (52)	51 (42)	
	III−IV	48 (52)	23 (45)	25 (61)		121 (55)	49 (48)	72 (58)	
IPI	0–2	62 (67)	35 (69)	27 (66)	0.83	112 (48)	56 (76)	56 (62)	0.091
	3–5	30 (33)	16 (31)	14 (34)		52 (52)	18 (24)	34 (38)	
IMPA1	Low	69 (75)	42 (82)	27 (66)	0.09	218 (94)	104 (97)	114 (90)	0.058
	High	13 (25)	9 (18)	14 (34)		15 (6)	3 (3)	12 (10)	
MAPK1	Low	27 (29)	12 (24)	15 (37)	0.25	50 (21)	27 (25)	23 (18)	0.205
	High	65 (71)	39 (76)	26 (63)		183 (79)	80 (75)	103 (82)	
PIP5K1A	Low	78 (85)	46 (90)	32 (78)	0.15	223 (96)	104 (97)	119 (94)	0.35
	High	14 (15)	5 (10)	9 (22)		10 (4)	3 (3)	7 (6)	
CACNG3	Low	77 (84)	46 (90)	31 (76)	0.09	131 (56)	63 (59)	68 (54)	0.508
	High	15 (16)	5 (10)	10 (24)		102 (44)	44 (41)	58 (46)	
RASGRP3	Low	82 (89)	47 (92)	35 (85)	0.33	183 (79)	77 (72)	106 (84)	0.026
	High	10 (11)	4 (8)	6 (15)		50 (21)	30 (28)	20 (16)	
SYK	Low	81 (88)	44 (86)	37 (90)	0.75	219 (94)	98 (92)	121 (96)	0.176
	High	11 (12)	7 (14)	4 (10)		14 (6)	9 (8)	5 (4)

**Table 2:** DE-miRNA target gene expression is associated with survival in DLBCL patients (*n* = 92) (log-rank test)

**Gene**	**Pathway**	**Targeting miRNA**	**OS** ^**a**^ **(** ***p*** **-val** **)**	**PFS (** ***p*** **-val)**
*PIP5K1A*	PI	miR-485-5p	<0.001	<0.001
*IMPA1*	PI	miR-381-3p	0.004	0.016
*SYK*	BCR	miR-370-3p	0.231	0.002
*RASGRP3*	BCR, MAPK	miR-409-3p, miR-485-5p	0.001	<0.001
*MAPK1*	MAPK	miR-370-3p, miR-409-3p, miR-493-5p, miR-485-5p	0.248	0.038
*CACNG3*	MAPK	miR-370-3p	0.224	0.007

^a^ *OS* overall survival, *PFS* progression-free survival, *PI* phosphatidylinositol, *BCR* B-cell receptor, *MAPK* mitogen-activated protein kinase

**Fig. 5:**
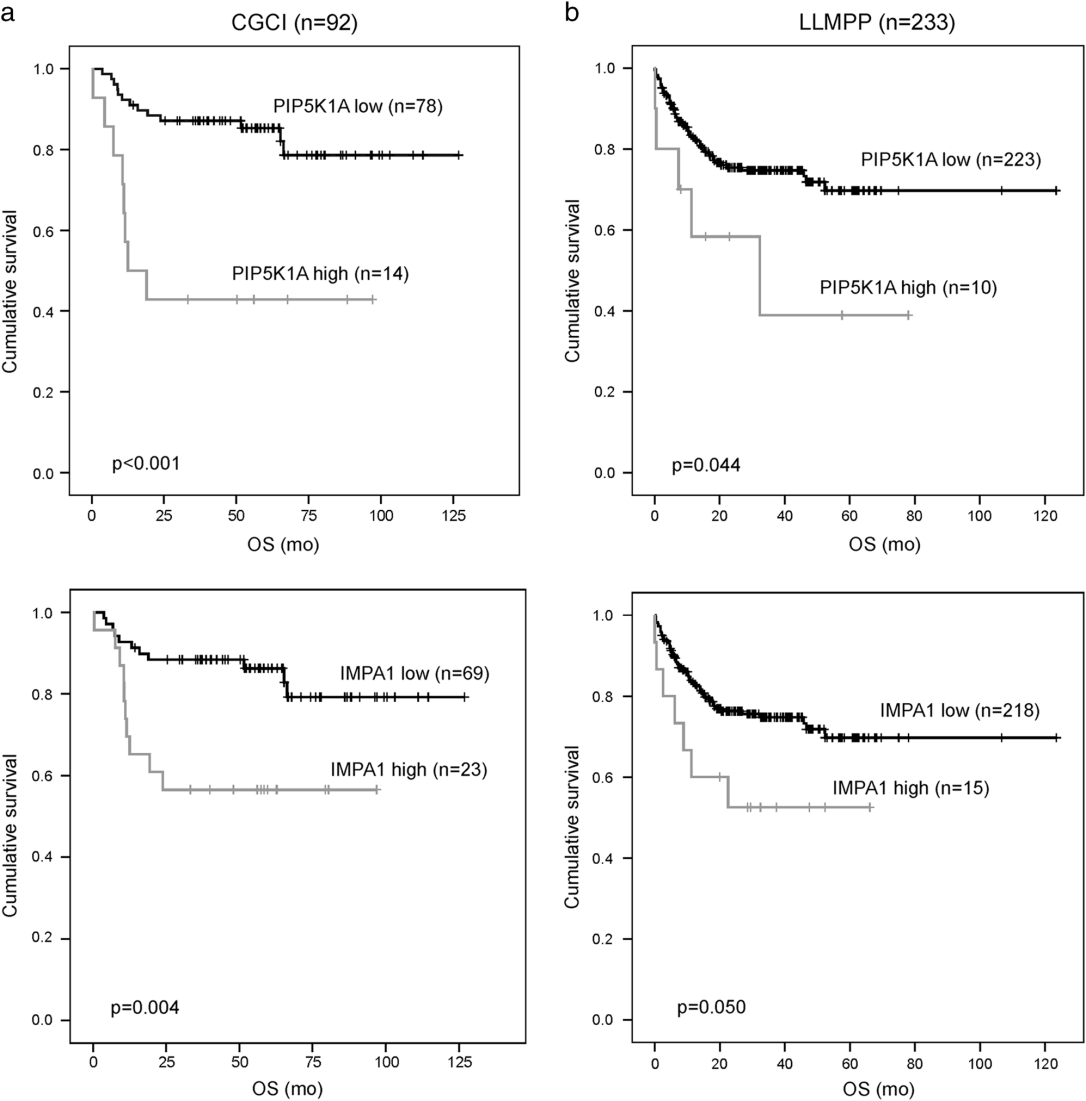
*PIP5K1A* and *IMPA1* are significantly associated with survival in two DLBCL patient cohorts **a**, **b** The chemoimmunotherapy-treated patients from two independent cohorts (CGCI *n* = 92 (**a**) and LLMPP *n* = 233 (**b**)) were divided into two groups with high and low gene expression. The ideal cut-off values were calculated using Cutoff Finder. Kaplan–Meier curves depict overall survival (OS) of patients whose tumors contained low or high levels of the selected genes. *p*-values were obtained using a log-rank test

To validate the survival data, LLMPP data set was exploited (*n* = 233) (Table 1)^3^. In this cohort, high expression of *IMPA1* and *PIP5K1A* was found to have adverse impact on OS (Fig. 5). Association of *PIP5K1A* with poor survival was restricted to samples of non-GCB subtype (not shown). Together, our clinical, molecular, and functional data show that differentially expressed miRNAs target gene expression and thereby regulate key cell survival pathways, proliferation of lymphoma cells and survival of patients with DLBCL progression.

## Discussion

Genomic profiling of paired primary-relapse samples enables the discovery of the biologic pathways and mechanisms that drive therapy resistance. Here, we have utilized NGS and profiled the miRNAome of matched primary and relapsed DLBCL. MiRNAs are important regulators of both normal and pathological cellular processes controlling gene expression through complex regulatory networks. Recently, it was reported that miRNAs are associated with DLBCL outcome and can be used as predictors of treatment effectiveness^10, 13^. To our knowledge, the present study is the first one characterizing the changes that occur in miRNA expression during the treatment course of DLBCL and validating these observations with functional studies. Our results show that miRNA expression profiles remain relatively constant as the disease progresses with only a few miRNAs that were differentially expressed between the primary-relapse pairs. The limitation of our study is the small sample size, which may hamper the identification of differences between primary and relapse tumors. However, the small set of differentially expressed miRNAs participates in the control of central lymphoma-associated pathways, and represents a set of potential molecular targets for therapeutic intervention. Putative targets for the differentially expressed miRNAs were enriched for important lymphoma-associated pathways, such as PI signaling system, BCR, and MAPK signaling. Specifically, they were targeted by miRNAs downregulated in relapse tissue, suggesting that the pathways are activated during DLBCL progression. Our data further demonstrated that high expression of selected genes from these signaling pathways in the pretreatment samples was associated with poor survival. Importantly, the expression of *PIP5K1A* and *IMPA1* genes had negative prognostic impact on survival in two independent DLBCL cohorts. *PIP5K1A* and *IMPA1* are components of the PI signaling pathway, which is an important intracellular second-messenger signaling system linking to the PI3K/AKT pathway. The PI3K/AKT pathway in turn plays an important role in controlling proliferation and survival of tumor cells, and therefore represents a promising therapeutic target in DLBCL^39^ *. IMPA1* (Inositol monophosphatase 1) is responsible for the provision of inositol required for synthesis of PI and polyphosphoinositides, whereas *PIP5K1A* (Phosphatidylinositol-4-Phosphate 5-Kinase, Type I, Alpha) catalyzes the phosphorylation of phosphatidylinositol 4-phosphate (PIP) to form phosphatidylinositol 4,5-bisphosphate (PIP_2_), implicated in a wide variety of cellular signaling pathways.

BCR signaling has also been suggested to act as a driver of lymphoma development^41^. It is essential for normal B-cell development and maturation, but has emerged as an important target for the treatment of B-cell malignancies, including DLBCL. Central hubs in the BCR signaling pathway include SYK, Bruton’s tyrosine kinase (BTK), and PI3K. Novel therapies targeting the BCR signaling in lymphoma are currently under investigation. For instance, ibrutinib, a small molecule inhibiting BTK has shown significant anti-tumor activity in clinical studies on B-cell lymphomas^42^. Additionally, tonic BCR signaling and lymphoma cell survival can be selectively targeted with a SYK inhibitor fostamatinib, which has shown activity against relapsed DLBCL in a phase I/II study^43^.

MiR-409-3p, miR-381-3p, and miR-370-3p were validated as being downregulated in the relapse samples of an independent patient cohort. Interestingly, overexpression of these miRNAs enhanced the chemosensitivity of DLBCL cells in vitro. Several studies have suggested that miRNAs are novel players in mediating drug resistance. Overexpression of miR-34a sensitized DLBCL cells to doxorubicin in vitro^44^. MiR-17~92 cluster mediated chemoresistance in mantle cell lymphoma^45^, and miR-331-5p and miR-27a were downregulated in doxorubicin-resistant leukemia cells^46^. MiR-381 may play a role in regulating the drug resistance in leukemia cells^47^.

In addition to differentially expressed miRNAs, we characterized miRNAs with high or low expression in the DLBCL samples compared to a control data set of non-malignant B-cells. Tumor suppressive miRNAs miR-129-5p, miR-663a, and miR-203a were hypermethylated at their promoter region, suggesting methylation as a potential mechanism for downregulation. This concurs with previous studies demonstrating hypermethylation of miR-129 and miR-203 in hematological cancers, including non-Hodgkin lymphoma^48, 49^. Indeed, lower miR-129-5p expression was associated with shorter survival in DLBCL patients both with and without R-CHOP treatment^31^. In our integrated miRNA−mRNA analysis miR-129-5p was predicted to target several oncogenes that included *IRF4, PIM1, FOXP1, SYK, BCL10, RUNX1*, and *ABL1*. These were enriched for cancer-associated pathways, such as regulation of I-kappaB kinase/NF-kappaB cascade, regulation of protein kinase cascade, cell death, and apoptosis (not shown).

In conclusion, our analysis of the miRNAome in matched primary refractory and relapsed DLBCL uncovers biological processes underlying relapse/progression. In addition, the data imply that miRNAs mediate the chemoresistance of DLBCL. The results are novel and promising and emphasize that the molecular mechanisms of DLBCL progression are complex, involve multiple pathways, and are heterogeneous between the patients.

## Electronic supplementary material

Supplementary Information

Table S4

Table S5

Table S6

Table S7
