# Supplementary material for: MicroRNAs regulate key cell survival pathways and mediate chemosensitivity during progression of diffuse large B-cell lymphoma
Source: Blood Cancer J. 2017 Dec 15;7(12):654. doi: 10.1038/s41408-017-0033-8 (PMC5802506; doi:10.1038/s41408-017-0033-8)
Supplement: Supplementary file 1 — Supplementary Information [file 41408_2017_33_MOESM1_ESM.pdf]

Supplementary Information

Leivonen et al. MicroRNAs regulate key cell survival pathways and mediate chemosensitivity during progression of diffuse large B-cell lymphoma

Supplementary Methods

RNA isolation

For the discovery cohort, total RNAs from fresh-frozen tissue samples were isolated with NucleoSpin TriPrep (Machery-Nagel, Düren, Germany). For the validation cohort, total RNAs were isolated with RecoverAll™ Total Nucleic Acid Isolation Kit for FFPE (Life Technologies, Thermo Fisher Inc., Waltham, MA, USA). Two or three 20 µM sections were cut from the FFPE blocks and processed according to the manufacturer's protocol. Total RNAs from the cell lines were isolated with MiRVana Total RNA Isolation kit (Life Technologies) according to the manufacturer's protocol.

Next-generation sequencing and data analysis

For miRNA sequencing, raw short reads were preprocessed by trimming the 3' adaptor sequences from raw short reads and removing unreliable reads with insufficient base quality or sequence length. Preprocessed reads were aligned to the human genome (NCBI37 v.70) and to known miRNA transcripts (miRBase v.20). Mapped reads were quantified and annotated to known mature miRNAs. One sample (DLBCL1_R) was filtered out during quality control due to poor sequence quality. We defined miRNAs to be expressed if at least 25% of the samples each had a minimum two mapped reads. Thereafter, expression was normalized to counts-per-million and log2-transformed.

Three miRNA subsets were identified based on mature miRNA expression across samples: those found to be differentially expressed between primary and relapse by using the R Bioconductor packages edgeR[1] and DESeq[2] with $p<0.05$; those belonging to the 20% of the highest expressed miRNAs in at least 50% of the samples (termed high expressed miRNAs); and those with non-zero expression in the bottom 50th percentile in more than 50% of samples (termed low expressed miRNAs). High and low expressed miRNAs were further filtered against similarly high and low expressed miRNAs in a reference dataset of normal and non-malignant human B cells downloaded from GEO (GSE15229). These analyses produced a set of miRNAs specifically and aberrantly expressed in lymphoma.

For total RNA sequencing, five sample pairs (DLBCL3, DLBCL4, DLBCL5, DLBCL6, DLBCL7; Supplementary Table S1) were used. Sample preparation and Illumina sequencing were performed by Beijing Genomics Institute. The total RNA libraries were constructed using a modified protocol of Illumina TruSeq total RNA library preparation kit which includes amplification with random primers and removal of ribosomal RNA with Ribo-Zero. Quality of RNA and the sequencing libraries were performed with Bioanalyzer 2100 instrument (Agilent technologies, Inc, CA, USA). The read depth was ~80 M per sample. Quality of the raw reads was assessed with FastQC. Trimomatic was used for adapter removal and low-quality base clipping. Alignments and expression quantification were obtained with Tophat and Cufflinks.

The Cancer Genome Characterization Initiative (CGCI) data were preprocessed as follows. Quality of the raw reads was assessed with FastQC. Trimomatic was used for adapter removal and low-quality base clipping. Alignments and expression quantification were obtained with Tophat and Cufflinks. From the 105 cases in the CGCI repository we included 92 patients who were treated with the R-CHOP regimen.

MiRNA promoter methylation analysis

Reduced representation bisulfite sequencing (RRBS)[3] was performed using Illumina HiSeq2000 (Illumina Inc., CA, USA) at the Institute of Molecular Medicine Finland on paired primary-relapse tumors from four DLBCL patients. Blood samples were available from two of these patients and they were used as normal tissue controls. Raw reads were quality and adapter trimmed with Trim_galore (http://www.bioinformatics.babraham.ac.uk/projects/trim_galore/), which uses Cutadapt[4] as a tool for adapter trimming. We used the RRBS mode in Trim_galore and set the quality trimming threshold to 28. The first 13 basepairs of the standard Illumina adapter were used for adapter trimming with stringency of 2. After quality and adapter trimming, we kept a read pair if both the read and the mate had a minimum length of 40.

The quality-controlled reads were then aligned to the human reference genome build hg19 using Bismark[5], and Bowtie2[6] was used as an internal aligner. The deduplication step was ignored since it is not applicable to RRBS. We used MethylExtract[7] for extracting methylation calls from the aligned bam files. MethylExtract quantifies methylation with considering single nucleotide substitutions which made it a favorable option. We used the options of ignoring the first 12 basepairs in each read since they are amenable to protocol bias, as well as discarding the second mate overlapping segment. We restricted the methylation calling to CpG context and combined the calls in both strands for each CpG dinucleotide in order to enhance the coverage. Finally, we only considered CpG sites with minimum coverage of 5. The resulted methylation calls for each CpG site were in the form of so-called beta values ranging between 0 (no methylation) to 1 (full methylation). To quantify methylation in the miRNA gene promoter, we computed the mean methylation of all CpGs in that region.

Immunoblotting

MiRNA-expressing SUDHL-4 cells were lysed in RIPA buffer. Equal amounts (40 μg) of protein lysates were fractionated on SDS-polyacrylamide gels and transferred to Trans-Blot Turbo nitrocellulose membranes (Bio-Rad). The membranes were blocked against non-specific binding using 5% skim milk and probed with specific antibodies for PI3Kγ p110 (PIK3CG; 5405, Cell Signaling Technologies, Danvers, MA, USA) and ERK1/2 (MAB1576, R&D Systems, Minneapolis, MN, USA). Equal loading was confirmed by probing the same membrane with an antibody for human β-actin (MA5-15739, Thermo Fisher Scientific, Rockford, IL, USA). The blots were visualized by enhanced chemiluminescence (ECL) detection system (Pierce, Thermo Fisher Scientific). Band densities were quantified by ImageJ software and normalized to β-actin.

References

1. Robinson MD, McCarthy DJ, Smyth GK. edgeR: a Bioconductor package for differential expression analysis of digital gene expression data. *Bioinformatics* 2010; **26**: 139-140.

2. Anders S, Pyl PT, Huber W. HTSeq--a Python framework to work with high-throughput sequencing data. *Bioinformatics* 2015; **31**: 166-169.

3. Gu H, Smith ZD, Bock C, Boyle P, Gnirke A, Meissner A. Preparation of reduced representation bisulfite sequencing libraries for genome-scale DNA methylation profiling. *Nat Protoc* 2011; **6**: 468-481.

4. Martin M. Cutadapt removes adapter sequences from high-throughput sequencing reads. *EMBnetjournal, North America* 2011; **17**.

5. Krueger F, Andrews SR. Bismark: a flexible aligner and methylation caller for Bisulfite-Seq applications. *Bioinformatics* 2011; **27**: 1571-1572.

6. Langmead B, Salzberg SL. Fast gapped-read alignment with Bowtie 2. *Nat Methods* 2012; **9**: 357-359.

7. Barturen G, Rueda A, Oliver JL, Hackenberg M. MethylExtract: High-Quality methylation maps and SNV calling from whole genome bisulfite sequencing data. *F1000Res* 2013; **2**: 217.

Supplementary tables

Table S1. Patient characteristics of the discovery and validation cohorts.

ID	Gender	Age	GC[a]/ non-GC	Stage	IPI	aaIPI	WHO (ECOG)	Treatment	TTF (mo)
Discovery cohort									
DLBCL1	M	62	GC	3	3	2	1	R-CHOEP+MTX+AraC	12.1
DLBCL2	F	64	GC	4	4	3	2	R-CHOEP+MTX+AraC	6.3
DLBCL3	F	74	non-GC	4	5	3	2	R-CHOP	9.1
DLBCL4	F	64	non-GC	4	4	2	1	R-CHOP	4.2
DLBCL5	F	76	GC	3	4	3	2	R-CHOP	62.2
DLBCL6	M	62	GC	4	4	3	3	R-CHOP+MTX	28.5
DLBCL7	M	69	GC	4	4	2	1	R-CHOP+AraC	12.4
Validation cohort									
DLBCL10	F	69	non-GC	3	3	3	2	R-CHOP	15.7
DLCBL11	M	49	GC	4	3	2	1	R-CHOP	19.7
DLBCL12	F	64	non-GC	4	4	3	4	R-CHOEP+AraC+MTX	61.0
DLBCL13	F	53	GC	4	nd	nd	2	R-CHOEP	40.7
DLBCL14	F	68	non-GC	3	nd	nd	nd	R-CHOP+M-BACOD	156.8
DLBCL15	M	81	GC	4	2	1	1	R-CHOP	28.7
DLBCL16	F	71	non-GC	4	4	3	3	R-CHOP+MTX	11.9
DLBCL17	M	78	non-GC	4	4	3	3	R-CHOP	12.3
DLBCL18	M	77	GC	3	3	2	1	R-CHOP	9.7
DLBCL19	F	74	GC	4	4	3	2	R-CHOP	11.9
DLBCL20	F	44	GC	3	4	3	1	R-CHOEP	12.3
DLBCL21	F	55	GC	4	4	3	3	R-CHOP	22.0
DLBCL22	F	71	non-GC	3	3	2	2	R-CHOP	17.0

[a]GC, germinal center; IPI, international prognostic index; aaIPI, age-adjusted IPI; TTF, time to treatment failure; MTX, methotrexate; AraC, cytosine arabinoside; nd, not determined; m-BACOD, methotrexate with leucovorin, bleomycin, doxorubicin, cyclophosphamide, vincristine, and dexamethasone

Table S2. MiRNAs having consistently low or high expression in DLBCL.

Low expressed miRNAs	High expressed miRNAs
hsa-miR-100-3p	hsa-let-7d-3p
hsa-miR-106a-3p	hsa-miR-106b-5p
hsa-miR-106b-3p	**hsa-miR-10a-3p**[a]
hsa-miR-10b-3p	**hsa-miR-10b-5p**
hsa-miR-1185-1-3p	**hsa-miR-125a-5p**
hsa-miR-1226-3p	**hsa-miR-125b-1-3p**
hsa-miR-1226-5p	**hsa-miR-125b-5p**
hsa-miR-1237-3p	hsa-miR-143-3p
hsa-miR-125a-3p	hsa-miR-146b-3p
hsa-miR-1271-5p	hsa-miR-150-3p
hsa-miR-127-5p	hsa-miR-182-3p
hsa-miR-129-5p	hsa-miR-193b-3p
hsa-miR-1303	hsa-miR-197-3p
hsa-miR-1306-5p	hsa-miR-21-3p
hsa-miR-133a-3p	hsa-miR-22-5p
hsa-miR-134-5p	hsa-miR-27b-3p
hsa-miR-138-5p	hsa-miR-361-5p
hsa-miR-140-5p	hsa-miR-500a-3p
hsa-miR-142-5p	hsa-miR-501-3p
hsa-miR-143-5p	hsa-miR-589-3p
hsa-miR-145-3p	hsa-miR-671-3p
hsa-miR-149-5p	hsa-miR-769-5p
hsa-miR-1587	hsa-miR-941
hsa-miR-16-1-3p	**hsa-miR-99b-5p**
hsa-miR-16-2-3p	
hsa-miR-16-5p	[a]miR-10 family members in bold
hsa-miR-183-5p	
hsa-miR-184	
hsa-miR-185-3p	
hsa-miR-187-5p	
hsa-miR-188-5p	
hsa-miR-18b-5p	
hsa-miR-1908-5p	
hsa-miR-1913	
hsa-miR-193a-5p	
hsa-miR-193b-5p	
hsa-miR-194-5p	
hsa-miR-199a-3p	
hsa-miR-19b-2-5p	
hsa-miR-19b-3p	
hsa-miR-200c-3p	
hsa-miR-203a	
hsa-miR-204-3p	
hsa-miR-20a-5p	

hsa-miR-20b-5p
hsa-miR-211-3p
hsa-miR-216b-5p
hsa-miR-218-1-3p
hsa-miR-219a-1-3p
hsa-miR-221-3p
hsa-miR-223-5p
hsa-miR-2277-5p
hsa-miR-23b-3p
hsa-miR-2467-3p
hsa-miR-25-5p
hsa-miR-26b-3p
hsa-miR-296-5p
hsa-miR-29b-1-5p
hsa-miR-29b-2-5p
hsa-miR-29c-5p
hsa-miR-3074-3p
hsa-miR-30b-5p
hsa-miR-3120-5p
hsa-miR-3138
hsa-miR-31-3p
hsa-miR-3144-5p
hsa-miR-3150b-3p
hsa-miR-3155b
hsa-miR-3161
hsa-miR-3162-5p
hsa-miR-3175
hsa-miR-3176
hsa-miR-3180
hsa-miR-3180-5p
hsa-miR-3184-5p
hsa-miR-3187-3p
hsa-miR-3197
hsa-miR-320c
hsa-miR-32-3p
hsa-miR-330-5p
hsa-miR-335-3p
hsa-miR-340-3p
hsa-miR-340-5p
hsa-miR-3622a-3p
hsa-miR-362-5p
hsa-miR-3652
hsa-miR-365a-5p
hsa-miR-3678-3p
hsa-miR-370-3p
hsa-miR-374a-3p

hsa-miR-374a-5p
hsa-miR-374b-3p
hsa-miR-378a-5p
hsa-miR-378b
hsa-miR-378f
hsa-miR-378h
hsa-miR-381-3p
hsa-miR-3909
hsa-miR-3922-3p
hsa-miR-3940-5p
hsa-miR-3944-5p
hsa-miR-3960
hsa-miR-409-5p
hsa-miR-410-3p
hsa-miR-411-5p
hsa-miR-429
hsa-miR-431-5p
hsa-miR-4326
hsa-miR-433-3p
hsa-miR-4435
hsa-miR-4451
hsa-miR-4488
hsa-miR-449c-3p
hsa-miR-4510
hsa-miR-4515
hsa-miR-4519
hsa-miR-4524a-5p
hsa-miR-4529-5p
hsa-miR-4537
hsa-miR-455-3p
hsa-miR-455-5p
hsa-miR-4638-5p
hsa-miR-4640-3p
hsa-miR-4649-3p
hsa-miR-4649-5p
hsa-miR-4654
hsa-miR-4661-5p
hsa-miR-4664-5p
hsa-miR-4675
hsa-miR-4685-5p
hsa-miR-4695-3p
hsa-miR-4707-5p
hsa-miR-4709-3p
hsa-miR-4728-3p
hsa-miR-4732-5p
hsa-miR-4748

hsa-miR-4751
hsa-miR-4758-5p
hsa-miR-4767
hsa-miR-4781-3p
hsa-miR-4786-3p
hsa-miR-4800-5p
hsa-miR-485-5p
hsa-miR-487b-3p
hsa-miR-493-3p
hsa-miR-493-5p
hsa-miR-5001-5p
hsa-miR-5010-3p
hsa-miR-505-3p
hsa-miR-5100
hsa-miR-511-5p
hsa-miR-543
hsa-miR-550a-3-5p
hsa-miR-550a-5p
hsa-miR-561-5p
hsa-miR-5698
hsa-miR-5701
hsa-miR-576-5p
hsa-miR-582-5p
hsa-miR-615-3p
hsa-miR-615-5p
hsa-miR-624-3p
hsa-miR-625-5p
hsa-miR-628-5p
hsa-miR-629-3p
hsa-miR-629-5p
hsa-miR-642a-5p
hsa-miR-654-3p
hsa-miR-654-5p
hsa-miR-663a
hsa-miR-7-5p
hsa-miR-766-3p
hsa-miR-767-3p
hsa-miR-873-5p
hsa-miR-874-3p
hsa-miR-937-3p
hsa-miR-9-3p

Table S3. Differentially expressed miRNAs between the primary and relapsed DLBCL.

miRNA ID	Relapse vs. primary	LogFC[a]	P-val	FDR
hsa-miR-5698	up	6.757	0.001	0.256
hsa-miR-485-5p	down	-3.792	0.001	0.256
hsa-miR-381-3p	down	-6.688	0.002	0.267
hsa-miR-33b-3p	up	6.870	0.005	0.483
hsa-miR-370-3p	down	-3.255	0.006	0.483
hsa-miR-409-3p	down	-2.475	0.008	0.574
hsa-miR-1226-5p	down	-6.902	0.011	0.659
hsa-miR-493-5p	down	-5.963	0.015	0.664
hsa-miR-4664-5p	down	-6.259	0.018	0.669
hsa-miR-3120-5p	down	-6.619	0.023	0.749
hsa-miR-550a-5p	up	7.214	0.029	0.749
hsa-miR-26b-5p	up	2.287	0.029	0.749
hsa-miR-223-5p	up	3.726	0.048	0.890

[a]FC, fold change; FDR, false discovery rate

Table S4. MiRNA-mRNA pairs for the high-expressed miRNAs.

Table S5. MiRNA-mRNA pairs for the low-expressed miRNAs.

Table S6. KEGG pathways significantly enriched among the targets of the low-expressed miRNAs.

Table S7. MiRNA-mRNA pairs for the differentially expressed miRNAs.

Please see separate excel files.

Table S8. KEGG pathways significantly (FDR<0.05) enriched among the targets of the differentially expressed miRNAs.

Name	p-value	FDR	Gene symbol
Leukocyte transendothelial migration	1.01E-08	1.31E-06	*PIK3R1, EZR, PIK3CG, CTNND1, RASSF5, GNAI3, PIK3CD, CYBB, RHOH, RAPGEF4,CLDN19*
Phosphatidylinositol signaling system	9.15E-07	5.95E-05	*DGKD, PIKFYVE, PIK3R1, PIK3CG, ITPKB, IMPA1, INPP5E, PIK3CD, SYNJ1, PIP5K1A, PIK3C2A, PLCD4*
Chronic myeloid leukemia	1.45E-05	0.000626	*MAPK1, STAT5B, PIK3R1, SMAD, PIK3CG, PIK3CD, MECOM, RUNX1, SHC4, CDKN2A, STAT5A*
Acute myeloid leukemia	7.88E-05	0.002562	*MAPK1, STAT5B, PIK3R1, PIK3CG, PPARD, PIK3CD, RUNX1, TCF7, STAT5A*
Colorectal cancer	0.00022	0.005717	*MAPK1, PIK3R1, SMAD3, PIK3CG, MAPK10, PIK3CD, TCF7*
Chagas disease	0.000336	0.007284	*PPP2R2B, CASP8, MAPK1, PIK3R1, SMAD3, PIK3CG, MAPK10, GNAI3, PIK3CD*
Jak-STAT signaling pathway	0.000456	0.008465	*IL22RA2, STAT5B, PIK3R1, PIK3CG, CCND3, IL4R, IL15RA, PIK3CD, STAM2, SPRED2, IL23A, CISH, STAT5A*
Cholinergic synapse	0.000707	0.009972	*KCNQ4, MAPK1, CHRM3, ADCY5, PIK3R1, PIK3CG, PRKACB, GNAI3, PIK3CD*
Pathways in cancer	0.000745	0.009972	*CASP8, MAPK1, STAT5B, PIK3R1, SMAD3, RET, PIK3CG, PPARD, FGFR2, RASSF1, MAPK10, RASSF5, PIK3CD, MECOM, RUNX1, TPM3, TCF7, TRAF1, CDKN2A, STAT5A*
B cell receptor signaling pathway	0.000798	0.009972	*MAPK1, PIK3R1, PIK3CG, NFATC2, SYK, PIK3CD, RASGRP3*
Hepatitis C	0.000867	0.009972	*PPP2R2B, LDLR, MAPK1, PIK3R1, SCARB1, PIK3CG, MAPK10, PPARA, PIK3CD, CLDN19, PDK1*
Toxoplasmosis	0.00092	0.009972	*CASP8, LDLR, MAPK1, PIK3R1, PIK3CG, MAPK10, GNAI3, PIK3CD, CD40, PDK1*
HTLV-I infection	0.001003	0.010033	*STAT5B, ADCY5, PIK3R1, SMAD3, ELK4, PIK3CG, PRKACB, NFATC2, CCND3, MAPK10, IL15RA, MYBL1, CHEK2, PIK3CD, CD40, CDKN2A, STAT5A*
Fc epsilon RI signaling pathway	0.001226	0.011089	*MAPK1, PIK3R1, PIK3CG, SYK, MAPK10, PIK3CD, PDK1*
Neurotrophin signaling pathway	0.001279	0.011089	*MAPK1, IRAK2, PIK3R1, BDNF, TP73, PIK3CG, MAPK10, PIK3CD, SHC4, PDK1*
ErbB signaling pathway	0.001385	0.011254	*MAPK1, STAT5B, PIK3R1, PIK3CG, MAPK10, PIK3CD, ERBB4, SHC4, STAT5A*
Non-small cell lung cancer	0.002266	0.017326	*MAPK1, PIK3R1, PIK3CG, RASSF1, RASSF5, PIK3CD, CDKN2A*
Viral carcinogenesis	0.003478	0.023905	*CASP8, MAPK1, STAT5B, HDAC11, PIK3R1, PIK3CG, PRKACB, CCND3, SYK, PIK3CD, TRAF1, CDKN2A, STAT5A, GTF2A2*
Fc gamma R-mediated phagocytosis	0.003494	0.023905	*MAPK1, PIK3R1, PIK3CG, SYK, ARPC5, PIK3CD, PIP5K1A*
Regulation of actin cytoskeleton	0.003732	0.024261	*MAPK1, CHRM3, PIKFYVE, PIK3R1, EZR, PPP1CA, PIK3CG, FGFR2, ARPC5, PIK3CD, PIP5K1A, PPP1CB*
MAPK signaling pathway	0.00428	0.026494	*MEF2C, CACNB2, CACNG3, MAPK1, BDNF, ELK4, FGFR2, PRKACB, NFATC2, MAPK10, MAP3K8, NF1, PPM1B, MECOM, RASGRP3, NLK*
Pancreatic cancer	0.004731	0.027955	*MAPK1, PIK3R1, SMAD3, PIK3CG, MAPK10, PIK3CD, CDKN2A*
Bacterial invasion of epithelial cells	0.005576	0.030833	*PIK3R1, PIK3CG, ARPC5, PIK3CD, SHC4*
Progesterone-mediated oocyte maturation	0.005692	0.030833	*MAPK1, ADCY5, PIK3R1, PIK3CG, PRKACB, MAPK10, GNAI3,PIK3CD*
Osteoclast differentiation	0.007003	0.036413	*MAPK1, PIK3R1, PIK3CG, NFATC2, SYK, MAPK10, TNFRSF11A, PIK3CD, CYBB*
p53 signaling pathway	0.007897	0.039484	*CASP8, RRM2B, TP73, CCND3, CHEK2, CDKN2A*
Insulin signaling pathway	0.008259	0.039764	*PRKAG2, MAPK1, PIK3R1, PPP1CA, PIK3CG, PRKACB, MAPK10, PIK3CD, SHC4, PPP1CB*

Supplementary figures

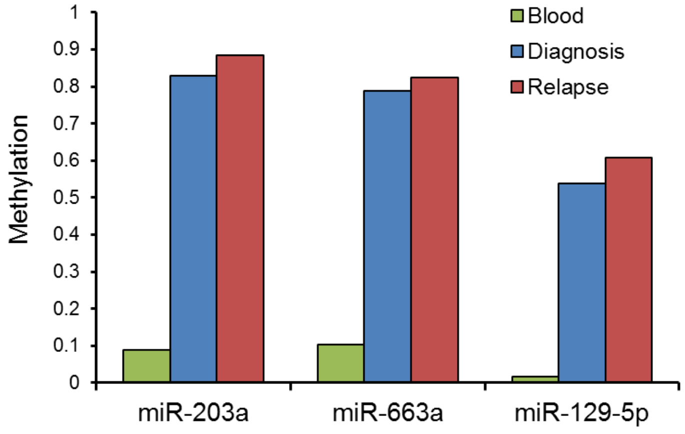

Figure S1. The degree of promoter methylation in the diagnosis and relapse DLBCL samples. Methylation was analyzed by Reduced Representation Bisulfite Sequencing (RRBS). The mean methylation scores for diagnosis (primary) and relapse samples are shown. Blood was used as control.

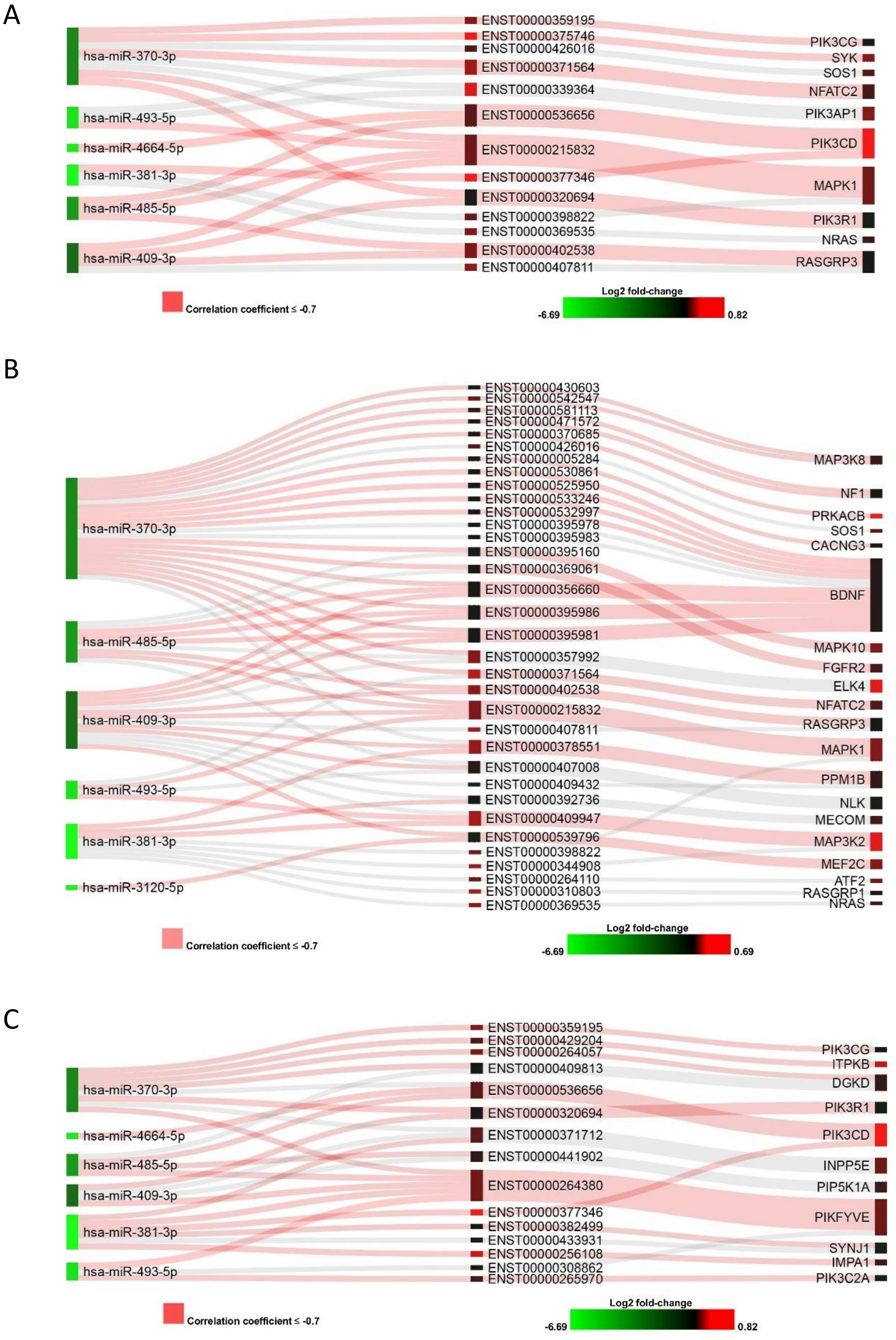

Figure S2. Sankey plots visualizing the miRNA-transcript interaction networks. A) B-cell receptor signaling pathway. B) MAPK signaling pathway. C) Phosphatidylinositol signaling system. The plots visualize miRNAs (left) targeting transcripts (middle) and their corresponding genes (right).

A

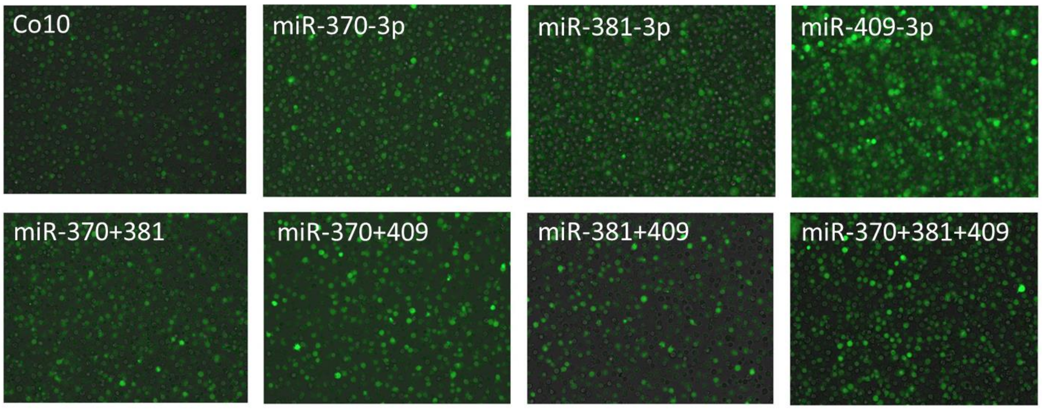

B

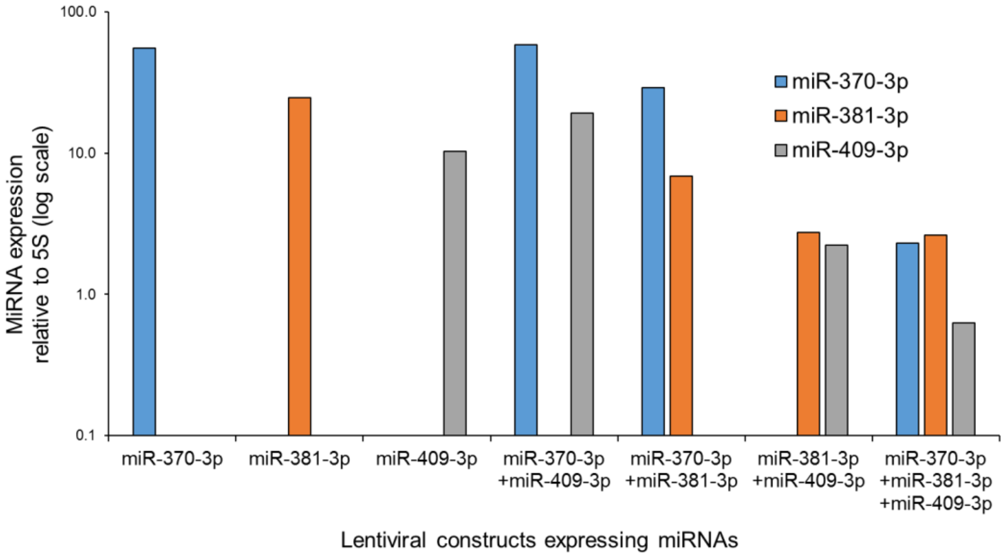

Figure S3. Characterization of the SU-DHL-4 cells stably expressing miRNAs. A) GFP expression of lentivirally transduced SU-DHL-4 cells was visualized under fluorescence microscope. Co10, non-targeting control. B) Relative miR-370-3p, miR-381-3p and miR-409-3p expression of the lentivirally transduced SU-DHL-4 cells was determined by qRT-PCR.

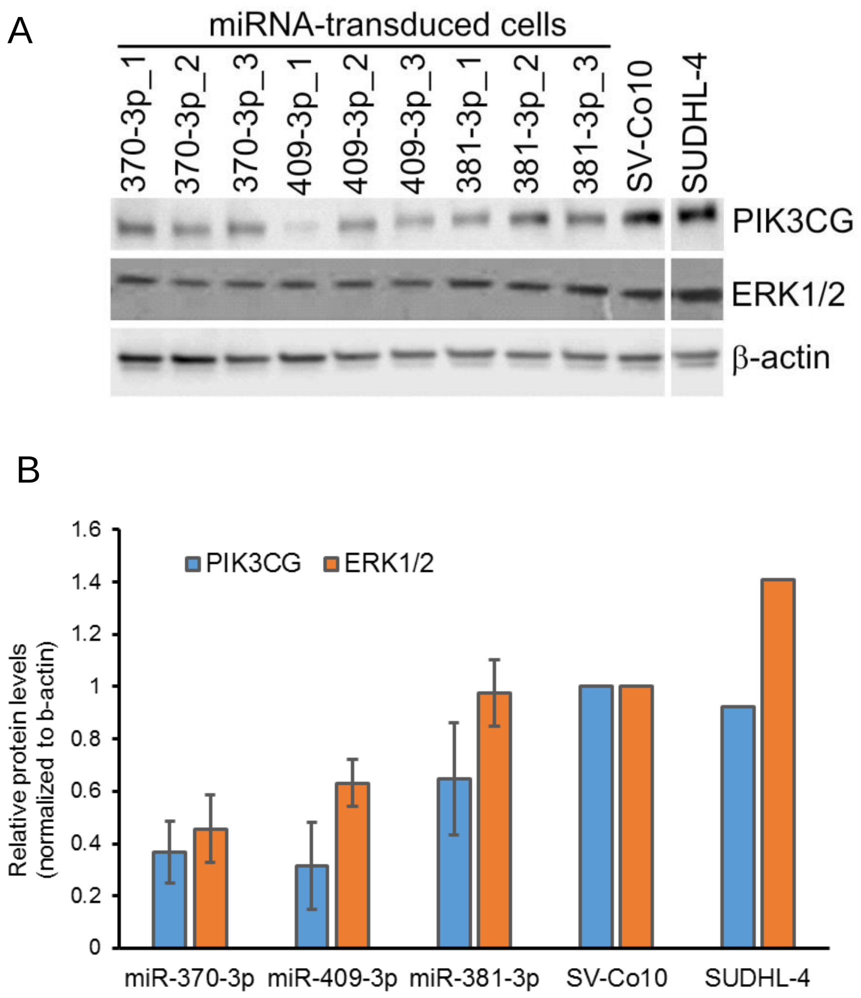

Figure S4. MiRNA-target gene validation by western blot. A) Three biological replicates of miR-370-3p, miR-409-3p, and miR-381-3p lentivirus transduced SUDHL-4 cells were analyzed by western blotting for the expression of PIK3CG and ERK1/2 proteins. SV-Co10 transduced SUDHL-4 cells and uninfected SUDHL-4 cells were used as controls. β-actin served as a loading control. B) Western blot image was quantified by ImageJ. The bars represent mean±SD compared to SV-Co10 transduced cells.

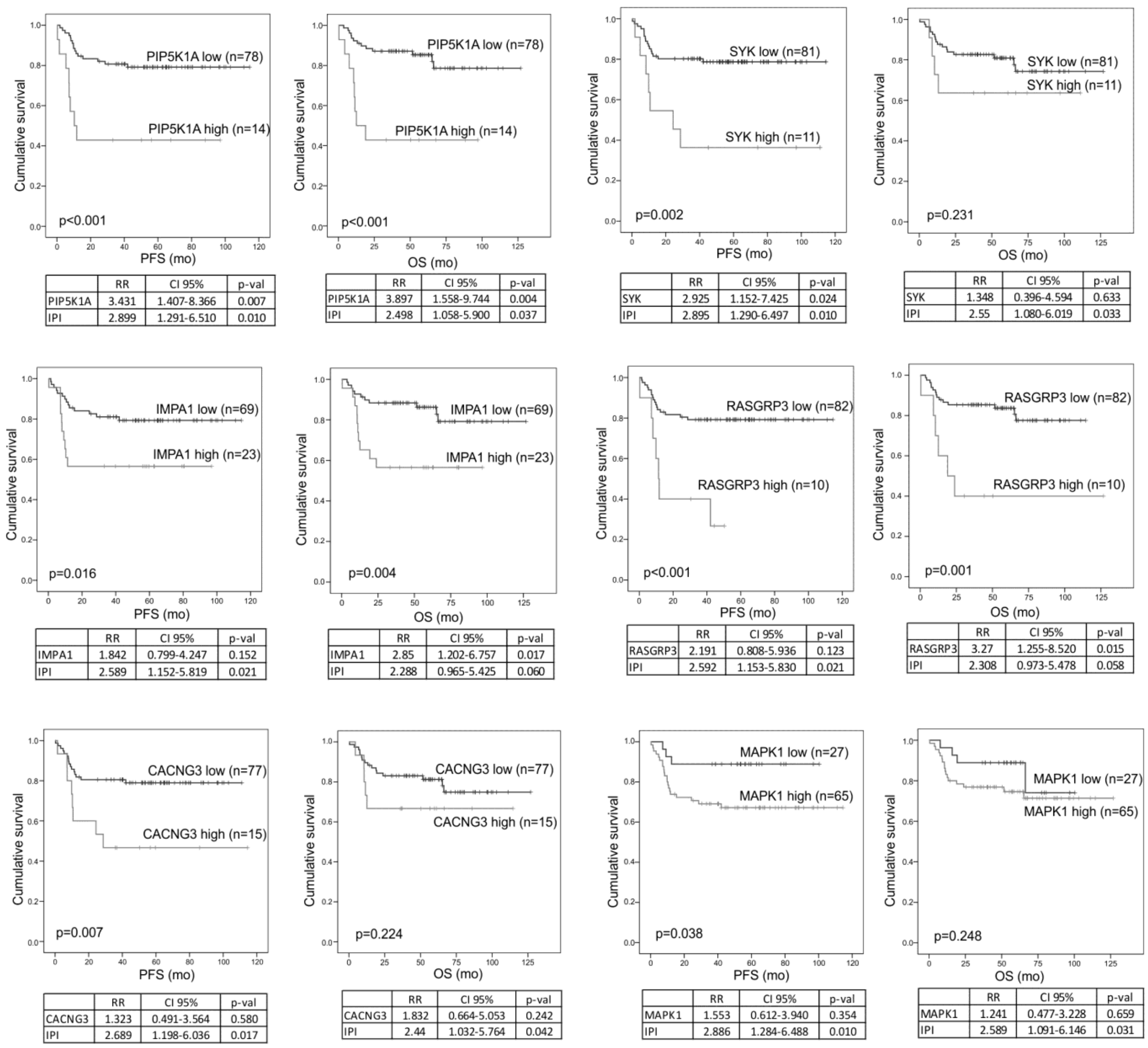

	RR	CI 95%	p-val
PIP5K1A	3.431	1.407-8.366	0.007
IPI	2.899	1.291-6.510	0.010

	RR	CI 95%	p-val
PIP5K1A	3.897	1.558-9.744	0.004
IPI	2.498	1.058-5.900	0.037

	RR	CI 95%	p-val
SYK	2.925	1.152-7.425	0.024
IPI	2.895	1.290-6.497	0.010

	RR	CI 95%	p-val
SYK	1.348	0.396-4.594	0.633
IPI	2.55	1.080-6.019	0.033

	RR	CI 95%	p-val
IMPA1	1.842	0.799-4.247	0.152
IPI	2.589	1.152-5.819	0.021

	RR	CI 95%	p-val
IMPA1	2.85	1.202-6.757	0.017
IPI	2.288	0.965-5.425	0.060

	RR	CI 95%	p-val
RASGRP3	2.191	0.808-5.936	0.123
IPI	2.592	1.153-5.830	0.021

	RR	CI 95%	p-val
RASGRP3	3.27	1.255-8.520	0.015
IPI	2.308	0.973-5.478	0.058

	RR	CI 95%	p-val
CACNG3	1.323	0.491-3.564	0.580
IPI	2.689	1.198-6.036	0.017

	RR	CI 95%	p-val
CACNG3	1.832	0.664-5.053	0.242
IPI	2.44	1.032-5.764	0.042

	RR	CI 95%	p-val
MAPK1	1.553	0.612-3.940	0.354
IPI	2.886	1.284-6.488	0.010

	RR	CI 95%	p-val
MAPK1	1.241	0.477-3.228	0.659
IPI	2.589	1.091-6.146	0.031

Figure S5. Survival association of selected miRNA target genes. The chemoimmunotherapy treated patients (n=92) were divided into two groups with high and low gene expression. The ideal cutoff values were calculated using using Cutoff Finder. Kaplan–Meier curves depict progression free survival (PFS) and overall survival (OS) of patients whose tumors contained low or high levels of the selected genes. P-values were obtained using a log-rank test. The panels below the Kaplan-Meiers show the results of multivariate analyses performed with Cox regression model. RR, risk ratio; CI, confidence interval.
